# Comparative transcriptome analysis of resistant and susceptible wheat in response to *Rhizoctonia cerealis*

**DOI:** 10.1186/s12870-022-03584-y

**Published:** 2022-05-10

**Authors:** Xingxia Geng, Zhen Gao, Li Zhao, Shufa Zhang, Jun Wu, Qunhui Yang, Shuhui Liu, Xinhong Chen

**Affiliations:** Shaanxi Key Laboratory of Genetic Engineering for Plant Breeding, College of Agronomy, Northwest A&F University, Yangling, 712100 Shaanxi China

**Keywords:** Comparative transcriptome, Plant–fungus interaction, Plant hormone, Sheath blight, *Rhizoctonia cerealis*, Wheat (*Triticum aestivum L.*)

## Abstract

**Background:**

Sheath blight is an important disease caused by *Rhizoctonia cerealis* that affects wheat yields worldwide. No wheat varieties have been identified with high resistance or immunity to sheath blight. Understanding the sheath blight resistance mechanism is essential for controlling this disease. In this study, we investigated the response of wheat to *Rhizoctonia cerealis* infection by analyzing the cytological changes and transcriptomes of common wheat 7182 with moderate sensitivity to sheath blight and H83 with moderate resistance.

**Results:**

The cytological observation showed that the growth of *Rhizoctonia cerealis* on the surface and its expansion inside the leaf sheath tissue were more rapid in the susceptible material. According to the transcriptome sequencing results, a total of 88685 genes were identified in both materials, including 20156 differentially expressed genes (DEGs) of which 12087 was upregulated genes and 8069 was downregulated genes. At 36 h post-inoculation, compared with the uninfected control, 11498 DEGs were identified in resistant materials, with 5064 downregulated genes and 6434 upregulated genes, and 13058 genes were detected in susceptible materials, with 6759 downregulated genes and 6299 upregulated genes. At 72 h post-inoculation, compared with the uninfected control, 6578 DEGs were detected in resistant materials, with 2991 downregulated genes and 3587 upregulated genes, and 7324 genes were detected in susceptible materials, with 4119 downregulated genes and 3205 upregulated genes. Functional annotation and enrichment analysis showed that the main pathways enriched for the DEGs included biosynthesis of secondary metabolites, carbon metabolism, plant hormone signal transduction, and plant–pathogen interaction. In particular, phenylpropane biosynthesis pathway is specifically activated in resistant variety H83 after infection. Many DEGs also belonged to the MYB, AP2, NAC, and WRKY transcription factor families.

**Conclusions:**

Thus, we suggest that the normal functioning of plant signaling pathways and differences in the expression of key genes and transcription factors in some important metabolic pathways may be important for defending wheat against sheath blight. These findings may facilitate further exploration of the sheath blight resistance mechanism in wheat and the cloning of related genes.

**Supplementary Information:**

The online version contains supplementary material available at 10.1186/s12870-022-03584-y.

## Background

Wheat (*Triticum aestivum* L.) is currently one of the most important food crops in the world, and wheat production is of great significance for ensuring food security and improving people's living standards. Wheat sheath blight is an important disease that detrimentally affects high and stable wheat yields throughout the world. In recent years, wheat sheath blight caused by *Rhizoctonia cerealis* has been aggravated in many parts of the world due to climate warming, leading to drastic economic losses that threaten food security [1]. *Rhizoctonia cerealis* is a necrotrophic fungus. Like many fungi, when *Rhizoctonia cerealis* infects the host, its hyphae grow tightly on the host surface. Before penetrating the host, the pathogenic fungi can form an infection cushion, a hyphal hoop, or single appressorium. Then, the infected hyphae produced by the base of the infestation pad or appressorium invade the host directly or through the stomata, and expand in the invaded cells, resulting in tissue lesions [2]. Some pathogenic fungi can even produce metabolites that are toxic to their hosts, which makes the hosts pathogenic [3–5]. In order to control pathogens, plants activate defense mechanisms, and then detect pathogens through cell surface and intracellular immune receptors. Plants recognize pathogen-associated molecular patterns (PAMP) through cell surface pattern recognition receptors and sense pathogen effectors by resistance protein, so as to produce PTI (PAMP triggered immunity) and ETI (effector triggered immunity) [6]. In addition, for the invasion of pathogenic bacteria, host plants will resist the infection of pathogenic microorganisms through many defense reactions, such as modification of host cell walls, release of reactive oxygen species (ROS), regulation of transcription factor genes and production of pathogenesis related proteins [7]. At present, our understanding of the interaction between the plant necrotrophic pathogen *Rhizoctonia cerealis* and wheat is very limited. Therefore, it is important to study the wheat sheath blight resistance mechanism in order to facilitate its prevention and control.

Phenylpropane biosynthesis pathway is one of the important secondary metabolic pathways in plants. It not only participates in the synthesis of important defense substances and secondary metabolites with defense function, lignin [8] and flavonoids [9], but also participates in the synthesis of important signal molecule salicylic acid (SA), which is closely related to plant disease resistance [10]. Lignin is a barrier to prevent the growth and reproduction of pathogens and an important defense means to resist the invasion and expansion of pathogens [11, 12]. Plant hormones and their signal transduction networks play an important role in plant resistance to pathogen infection [13, 14]. A large number of studies have shown that SA and JA play a key role in the interaction of plant pathogens [15, 16]. Hormone signal transduction is inseparable from the regulation of transcription factors. As pathway regulating genes, plant transcription factors play an important role in plant disease resistance response [17–19]. MYB, NAC, AP2 and WRKY family transcription factors are involved in JA signaling pathway. These transcription factors and their homologues may bind to JAZs and regulate specific JA response [20–23]. MYB, NAC, AP2 and WRKY family transcription factors are involved in JA signaling pathway. These transcription factors and their homologues may bind to JAZs and regulate specific JA response [21–26]. Moreover, the expression of NAC transcription factors is also induced by pathogen infection and plays a role in plant resistance to necrotizing pathogens [24, 25]. WRKY transcription factor also responds to PTI response in MAPK signal cascade pathway and interacts with other proteins in the family to jointly regulate plant response to pathogens [18]. Moreover, the expression of NAC transcription factors is also induced by pathogen infection and plays a role in plant resistance to necrotizing pathogens [27, 28]. WRKY transcription factor also responds to PTI response in MAPK signal cascade pathway and interacts with other proteins in the family to jointly regulate plant response to pathogens [19].

In the research of plant disease resistance, due to the advantages of transcriptome sequencing in data mining and mechanism analysis, this technology is widely used to explore the interaction between host plants and pathogens [26]. With the help of transcriptome technology, the gene information of plant interaction with pathogens can be deeply analyzed, so as to provide an important data basis for the mining of plant disease resistance related genes and the analysis of corresponding resistance mechanism [27, 28]. Up to now, a large number of studies on plant transcriptome infected by pathogens have been reported, and the molecular mechanism of resistance and the regulation of gene expression have been discussed. Kawahara et al. (2012) used RNA-Seq technology to perform mixed transcriptional sequencing of rice and *Magnaporthe oryzae* interaction, and found that the expression of pathogenesis-related and phytoalexin biosynthetic genes were upregulated in rice [29]. A total of 3258 DEGs were obtained by transcriptome sequencing of wheat roots aseptically cultured and inoculated by *Gaeumannomyces graminis var. tritici*, and a series of possible pathogenic factors of wheat total erosion were screened [30]. Zhang et al. (2017) analyzed the gene expression changes induced by rice AG1 IA strain *Rhizoctonia solani* at different inoculation time points by RNA-seq. By comparing the transcriptome data of moderately resistant variety and sensitive variety, 4802 DEGs and some metabolic pathways that play an important role in disease resistance were identified [31].

In recent years, comparative transcriptome sequencing has been used widely to study the mechanisms associated with interactions between plants and pathogens [30–33]. The response of wheat to fungal infection is highly complex, where it involves a series of biological reactions and physiological processes. In the present study, in order to understand the sheath blight resistance mechanism in wheat and the changes at the molecular level in different resistant materials after *Rhizoctonia cerealis* infection, we analyzed the responses of two types of wheat at two time points after *Rhizoctonia cerealis* infection by using RNA-Seq. The two plant materials comprised wheat H83 with moderate resistance to *Rhizoctonia cerealis* and wheat 7182 with moderate susceptibility to *Rhizoctonia cerealis*. To the best of our knowledge, this is the first study to apply comparative transcriptome analysis to explore the gene expression patterns in response to *Rhizoctonia cerealis* infection in resistant and susceptible wheat materials. Comparisons of the RNA-Seq data obtained for the resistant and susceptible materials detected significant differences in the expression of genes in defense signaling pathways and metabolic pathways in response to *Rhizoctonia cerealis* infection. We identified candidate genes associated with resistance to sheath blight in wheat, which may facilitate further exploration of wheat–*Rhizoctonia cerealis* interactions.

## Results

### Scanning electron microscopy observations of leaf sheath infection by *Rhizoctonia cerealis*

**Fig. 1:**
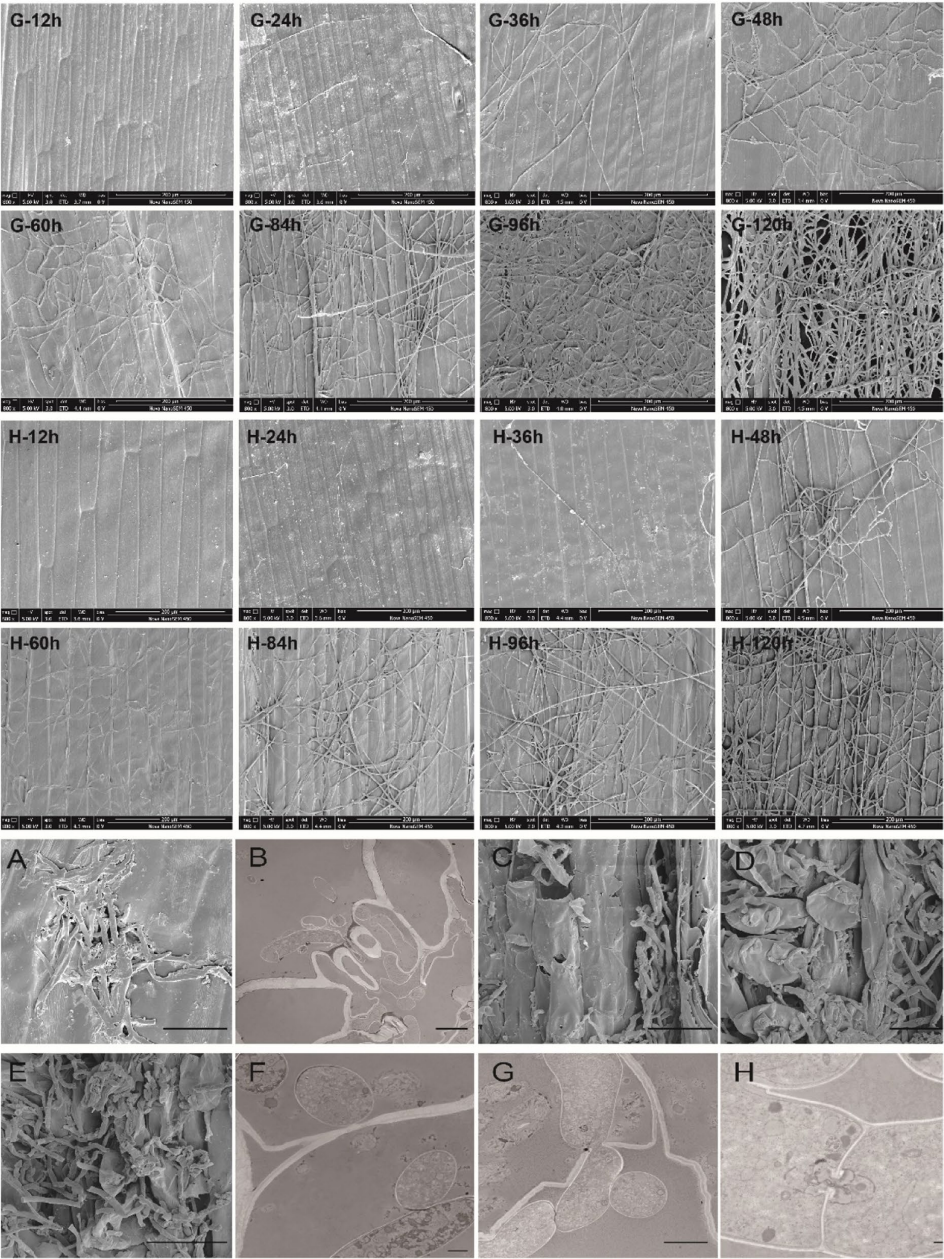
Infection of hypha in inoculated leaf sheath in resistant susceptible material H83 and susceptible material 7182 at different time point. G-12h-G-120h: hypha growth on leaf sheath of susceptible material 7182 at different time points after inoculation; H-12h-H-120h: hypha growth on leaf sheath of susceptible material 7182 at different time points after inoculation; A-H: electron microscopic image of hyphae invading leaf sheath; Bar: (G-12h-H-120h): 200 μm; (A, C, D, E): 100 μm; B: 5 μm; F: 2 μm; G:5 μm; H:1 μm

In order to investigate the invasion of wheat leaf sheaths by *Rhizoctonia cerealis*, we used *Rhizoctonia cerealis* to inoculate a derived progeny line of *Psathyrostachys huashanica* Keng called H83 and its parent line 7182. The leaf sheaths were observed by scanning electron microscopy at the inoculation site and at different times post-inoculation. At 12 h post-inoculation, no hyphae were observed on the surfaces of the leaf sheaths (Fig. 1, G-12h, H-12h). At 24 h post-inoculation, small numbers of hyphae were visible on the surfaces of the sheath cells (Fig. 1, G-24h, H-24h). At 36 h post-inoculation, mycelium with obvious branches was observed in the susceptible material (Fig. 1, G-36h). Compared with the susceptible material, the mycelium developed slowly with a small number of branches on the surface of the resistant material (Fig. 1, H-36h). At 48 h-120h post-inoculation, compared with the susceptible material, the growth of hyphae was slower on the surface of the resistant material, with fewer hyphal branches and a sparser hyphal network (Fig. 1, G-48h, H-48h, G-60h, G-84h, G-96h, G-120h, H-60h, H-84h, H-96h, H-120h). Moreover, the invasive mycelium was observed in cross sections of the leaf sheaths at 84 h, 96 h, and 120 h post-inoculation (Fig. 1 C, D, E). Thus, we conclude that the mycelium growth rate was slower on the surface of the leaf sheath in the resistant material compared with the susceptible material.

### Observation of leaf sheath infection by *Rhizoctonia cerealis* (transverse section of leaf sheath)

**Fig. 2:**
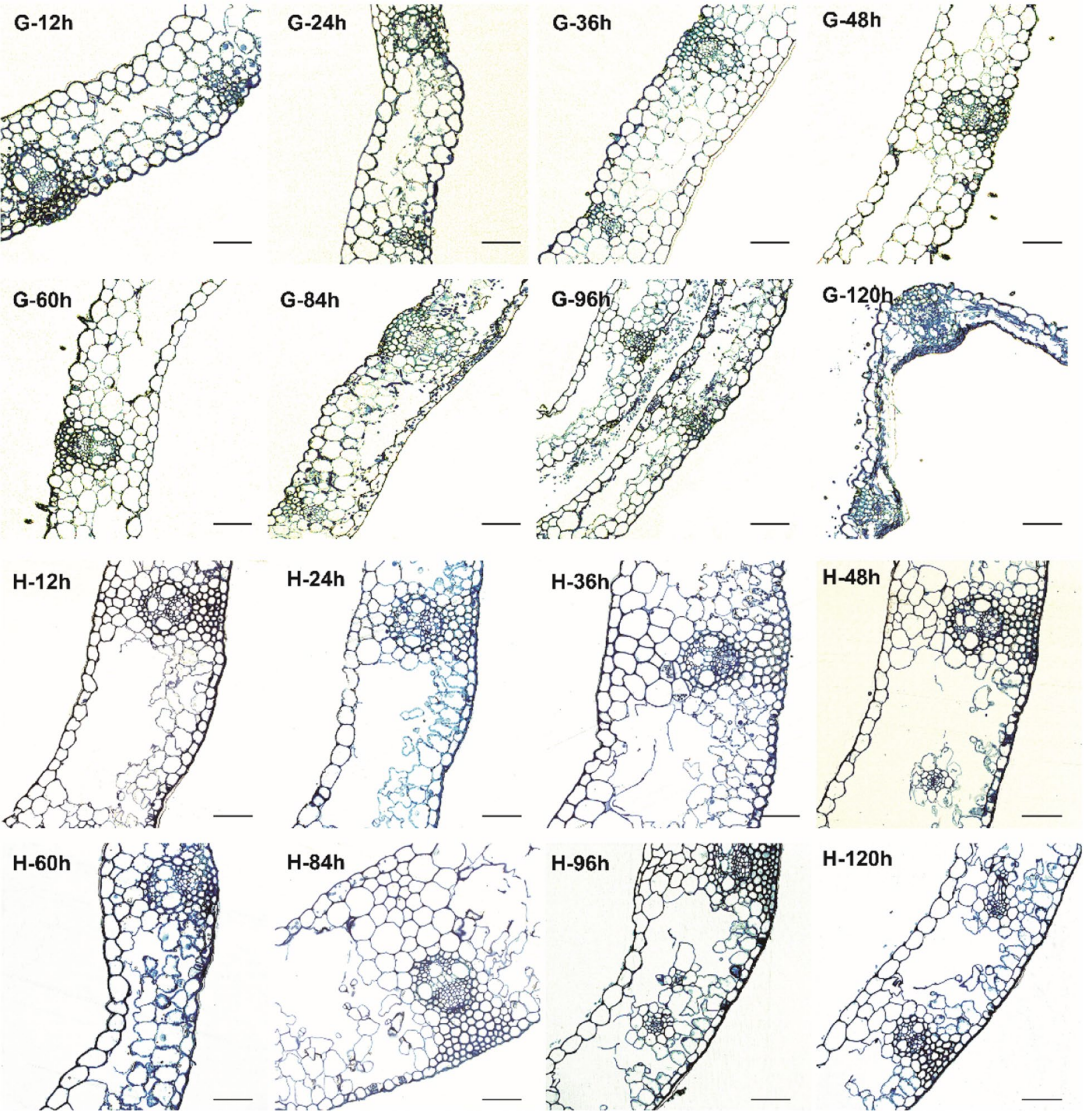
Images of sheath blight mycelium infection of leaf sheaths at different times after inoculation. G-12h-G-120h: hypha growth on leaf sheath of susceptible material 7182 at different time points after inoculation; H-12h-H-120h: hypha growth on leaf sheath of susceptible material 7182 at different time points after inoculation. Bar: 50μm

In order to further understand the specific invasion process by *Rhizoctonia cerealis* in the leaf sheaths, we observed half-thin and ultra-thin sections of the leaf sheaths at different times post-inoculation (Fig. 1B, F, G, H and 2). Invasive mycelium was not observed in the leaf sheath cells of the resistant or susceptible materials from 12 h to 48 h post-inoculation (Fig. 2, G-12h, G-36h, G-48h, H-12h, H-24h, H-36h). At 60h post-inoculation, a small amount of mycelium had entered the sheath epidermal cells in the disease-resistant material (Fig. 2, H-60h). However, it was not observed in the susceptible materials (Fig. 2, G-60h). At 84 h post-inoculation, the hyphae invaded the sheath cells in the susceptible material and diffused widely around the xylem cells (Fig. 2, G-84h). At this time, the mycelium was still in the epidermal cells of leaf sheath in the resistant material (Fig. 2, H-84h). At 96 h-120h post-inoculation, the hypha continued to expand. The leaf sheath cells were obviously deformed due to the severe infection by hyphae (Fig. 2, G-96h, G-120h). However, in the disease-resistant material, after repeated sectioning and careful observations, the hyphae that invaded the epidermal cells of the leaf sheath in the previous stage were not observed in this stage (Fig. 2, H-96h, H-120h). Thus, we conclude that the expansion of the mycelium was hindered in the resistant material.

In addition, we observed that the hyphae entered the leaf sheath through the stomata at different times post-inoculation (Fig. 1 A, B), thereby indicating that the infection of pathogenic hyphae directly through the stomata is important for the wheat sheath blight disease process. After hyphae invaded the leaf sheath tissue in the susceptible material, we observed the expansion of the hyphae between cells. First, the hyphae were close to the cell wall and the cell wall then became increasingly thinner. The hyphae formed a penetration peg through the dissolved cell wall, and finally entered the adjacent cells (Fig. 1 F, G). We also observed the unique barrel-shaped structure of the fungus (Fig. 1 H). Thus, the growth of *Rhizoctonia cerealis* on the surface and its expansion inside the leaf sheath tissue were more rapid in the susceptible material, and the time required for the mycelium to invade the leaf sheath cells was 60–84 h post-inoculation.

### Transcriptome sequencing analysis

**Fig. 3:**
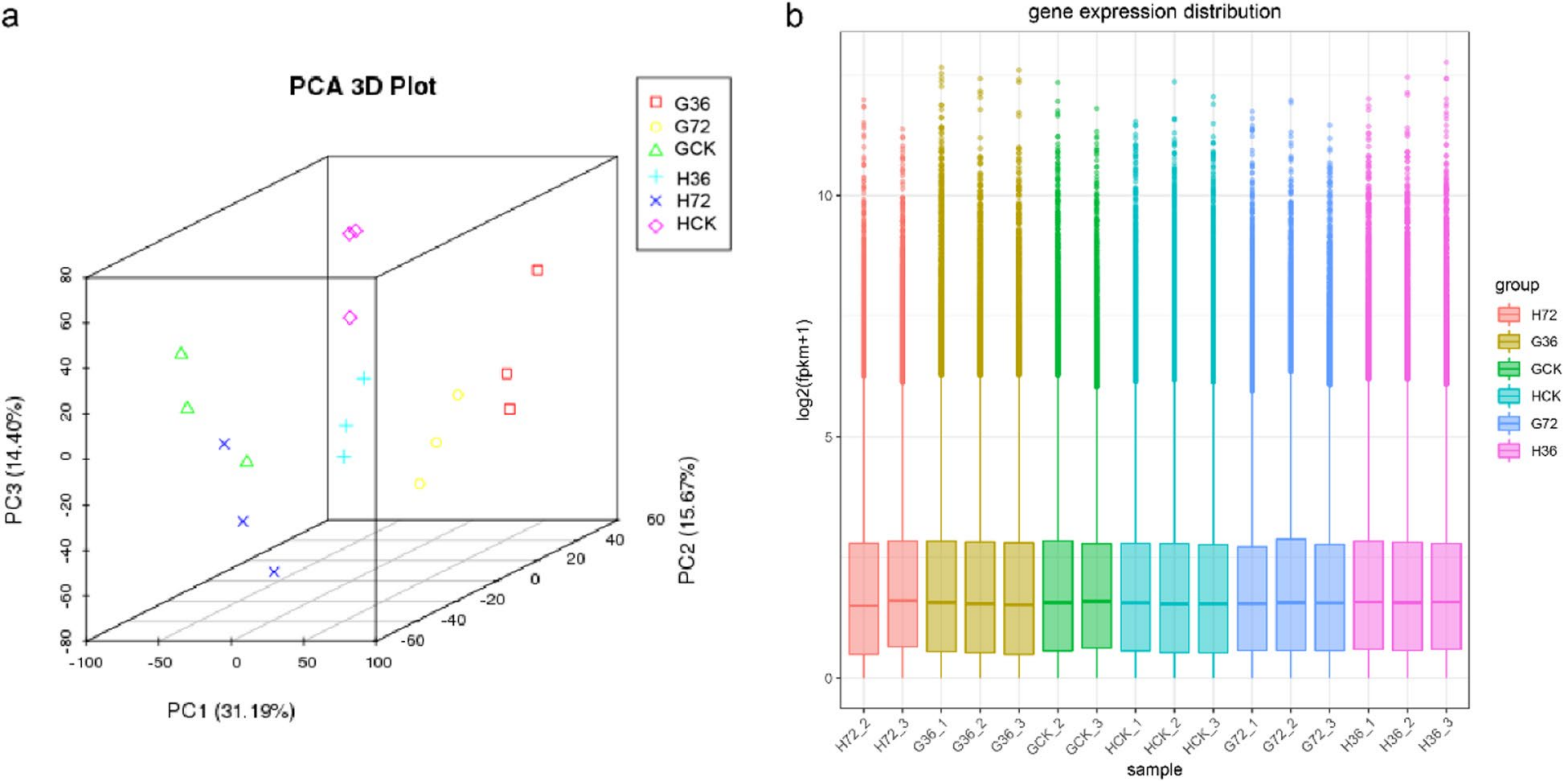
Principal component analysis of samples (**a**). Distribution of expressed genes in each sample (**b**). The horizontal axis represents each sample and the vertical axis represents the fragments per kilobase of transcripts per million mapped reads (FPKMs) values for different samples

In order to understand the mechanism associated with wheat sheath blight resistance and to identify resistance-related genes, RNA-Seq was used to analyze the gene expression profiles for H83 and 7182, and the DEGs were screened using bioinformatics techniques. The cytological observation showed that 36 h post-inoculation of *Rhizoctonia cerealis* was the time for the hyphae to colonize stably on the surface of leaf sheath, and it was also the time the growth of hyphae was significantly different on the surface of leaf sheath of the two materials before the hyphae entered the leaf sheath cells. At 60h post-inoculation, the hyphae were still on the surface of the leaf sheath of the susceptible material, and 84h post-inoculation had completely invaded the interior of the leaf sheath cells and expanded between the leaf sheath cells. Therefore, we speculate that 72h post-inoculation may be the critical time point for the *Rhizoctonia cerealis* to invade the susceptible material. Based on the above results, we took 36 h and 72 h post-inoculation as the sampling time points for transcriptome sequencing. The leaves corresponding to the inoculated leaf sheath (i.e. counting the second leaf from the base of wheat stem) of the resistant material H83 (HCK, H36, and H72 indicate leaf samples from the resistant material H83 at 0 h, 36 h, and 72 h post-inoculation, respectively) and susceptible material 7182 (GCK, G36, and G72 indicate leaf samples from the susceptible material 7182 at 0 h, 36 h, and 72 h post-inoculation, respectively) were sampled at 0 h, 36 h, and 72 h post-inoculation for transcriptome sequencing. Three biological replicates were performed for each sample with a total of 18 samples (HCK-1, HCK-2, HCK-3, H36-1, H36-2, H36-3, H72-1, H72-2, H72-3, GCK-1, GCK-2, GCK-3, G36-1, G36-2, G36-3, G72-1, G72-2, and G72-3). In total, 200 GB of clean bases were generated. After filtering the data for each sample, 11.1 GB of high-quality clean bases were obtained on average when the base percentage of Q20 (sequencing error rate = 3%) exceeded 96.58%. The GC contents of all the identified bases ranged between 55.16% and 56.82%. Principal component analysis showed that except for the overlap of samples H72-1 and GCK-1, the biological repeated data of other samples were clustered and separated according to time point, processing and genotype, indicating that the experimental processing was effective (Fig. 3 a). In order to ensure the analysis quality of data, we removed the overlapping samples H72-1 and GCK-1 for subsequent data analysis. The box plots for each sample library suggested that the differences in the distributions were low between the three repeated libraries of each sample (Fig. 3 b). Therefore, the results indicated that the quality of the data obtained by sequencing was reliable and they were suitable for subsequent analyses.

### DEGs in response to *Rhizoctonia cerealis*

**Fig. 4:**
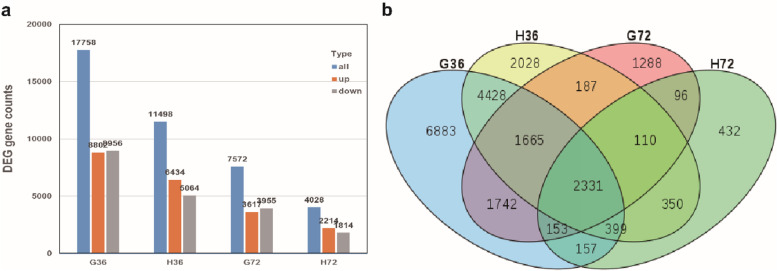
Histogram (**a**) and Venn diagram (**b**) based on the DEGs in H83 and 7182 after *Rhizoctonia cerealis* infection at the different time points. The horizontal axis represents different samples, the the vertical axis indicates the number of DEGs. The blue column, brick red column and gray column represent the total DEGs, up-regulated DEGs and down-regulated DEGs, respectively. Each circle represents all differentially expressed genes expressed in one sample

We used the DESeq2 R package (v1.16.1) to screen the DEGs between samples, where we applied | log_2_foldchange (FC) | > 1 and corrected error detection rate Padj < 0.01 as the standards for DEG screening. In order to determine genes with changes in their expression levels and the stages when these gene changes occurred, we compared materials collected after different times post-inoculation with a control of that genotype. As a result, a total of 88685 genes were identified in the two materials. Comparisons of the DEGs in the two materials at different times post-inoculation are presented in Fig. 4 a (details in Additional file 1: Table S1). At 36 h post-inoculation, 17758 DEGs (8802 upregulated and 8956 downregulated) were identified in the susceptible material and 11,498 DEGs (6434 upregulated and 5064 downregulated) in the resistant material. At 72 h post-inoculation, the number of DEGs decreased to 7572 (3617 upregulated and 3955 downregulated) in the susceptible material and 4028 (2214 upregulated and 1814 downregulated) in the resistant material. The results showed that when the two wheat materials were infected by *Rhizoctonia cerealis*, wheat material 7182 needed to mobilize more gene differential expression in response to infection than H83.

Venn diagram analysis based on the DEGs in the two materials after inoculation showed that at 36 h post-inoculation, the number of DEGs was 11498 in the resistant material H83, where 2028 were specific expression sites in H83, 3996 were common DEGs, 4428 were DEGs in H83 but they were also expression sites in material 7182, 749 were common DEGs in both materials at 72 h post-inoculation. The number of DEGs was 17758 in the susceptible material 7182, where 6883 were specific expression sites in the susceptible material 7182, 4428 were expression sites in material 7182 but also DEGs in H83, 3996 were common DEGs, and 2451 were common DEGs in the two materials at 72 h post-inoculation. At 72 h post-inoculation, the number of DEGs decreased in the two materials with 4028 DEGs in H83, where 1288 were unique expression sites in H83, 3441 were common DEGs, 96 were DEGs in H83 but they were also expressed in material 7182, 3704 were common DEGs in both materials at 36 h post-inoculation. We found 7572 DEGs in susceptible material 7182, where 432 were specific expression sites in 7182, 96 were expression sites in 7182 but also DEGs in H83, 2441 were common DEGs, and 1059 were common DEGs in both materials at 36 h post-inoculation (Fig. 4 b). the unique DEGs in a single material and the common differentially expressed genes between the two materials may be the cause of their differences in resistance when they were infected by *Rhizoctonia cerealis*.

### Functional classification of DEGs

**Fig. 5:**
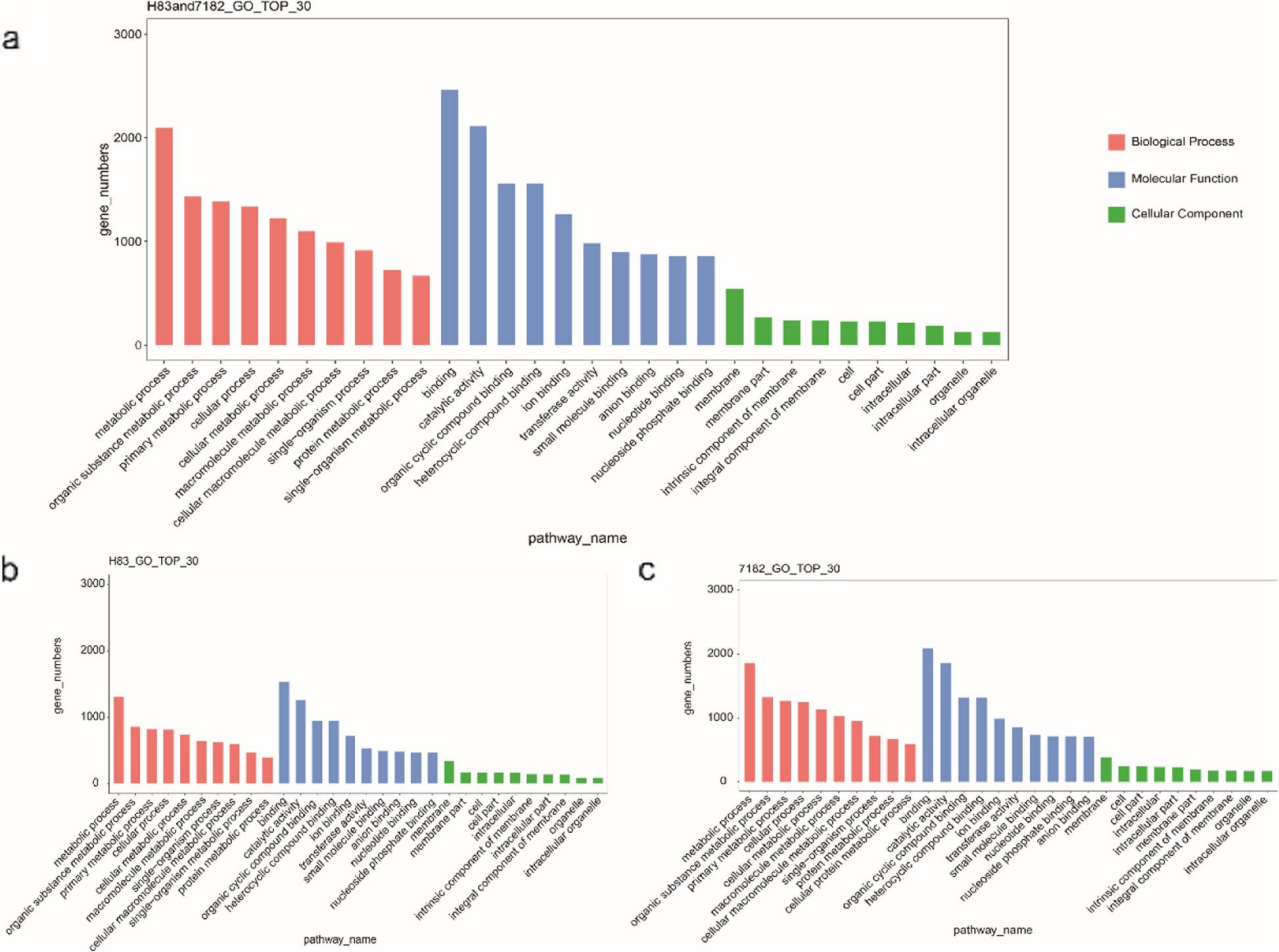
GO enrichment analysis of DEGs in resistant material H83 and susceptible material 7182 at different times after inoculation. The x-axis represents the GO term for the annotated DEG and the y-axis represents the number of DEGs annotated for each GO term. (**a**–**b**) GO enrichment analysis of DEGs in susceptible material 7182 at 36 and 72 h post-inoculation, respectively. (**c**–**d**) GO enrichment analysis of DEGs in resistant material H83 at 36 and 72 h post-inoculation, respectively

In order to screen the resistance-related genes to sheath blight, we classified the common DEGs of the two materials and the unique DEGs of the two materials. Blast2GO was used to classify the DEGs. The 11274 common DEGs in the two materials were annotated to 30 functional items (Fig. 5 a; details in Additional file 2: Table S2). The most representative term in biological processes was “metabolic process”, in cellular components was “membrane”, and in molecular functions was “binding”. The 3503 DEGs specific to disease resistant material H83 were annotated to 30 functional items (Fig. 5b; details in Additional file 2: Table S2). the most representative biological processes, cellular components, molecular functions were “metabolic process”, “membrane”, “catalytic activity” separately. The 7472 DEGs specific to susceptible material 7182 were annotated to 30 functional items (Fig. 5 c; details in Additional file 2: Table S2). The most representative biological processes, cellular components and molecular functions were “metabolic process”, “membrane” and “ion binding” separately.

**Fig. 6:**
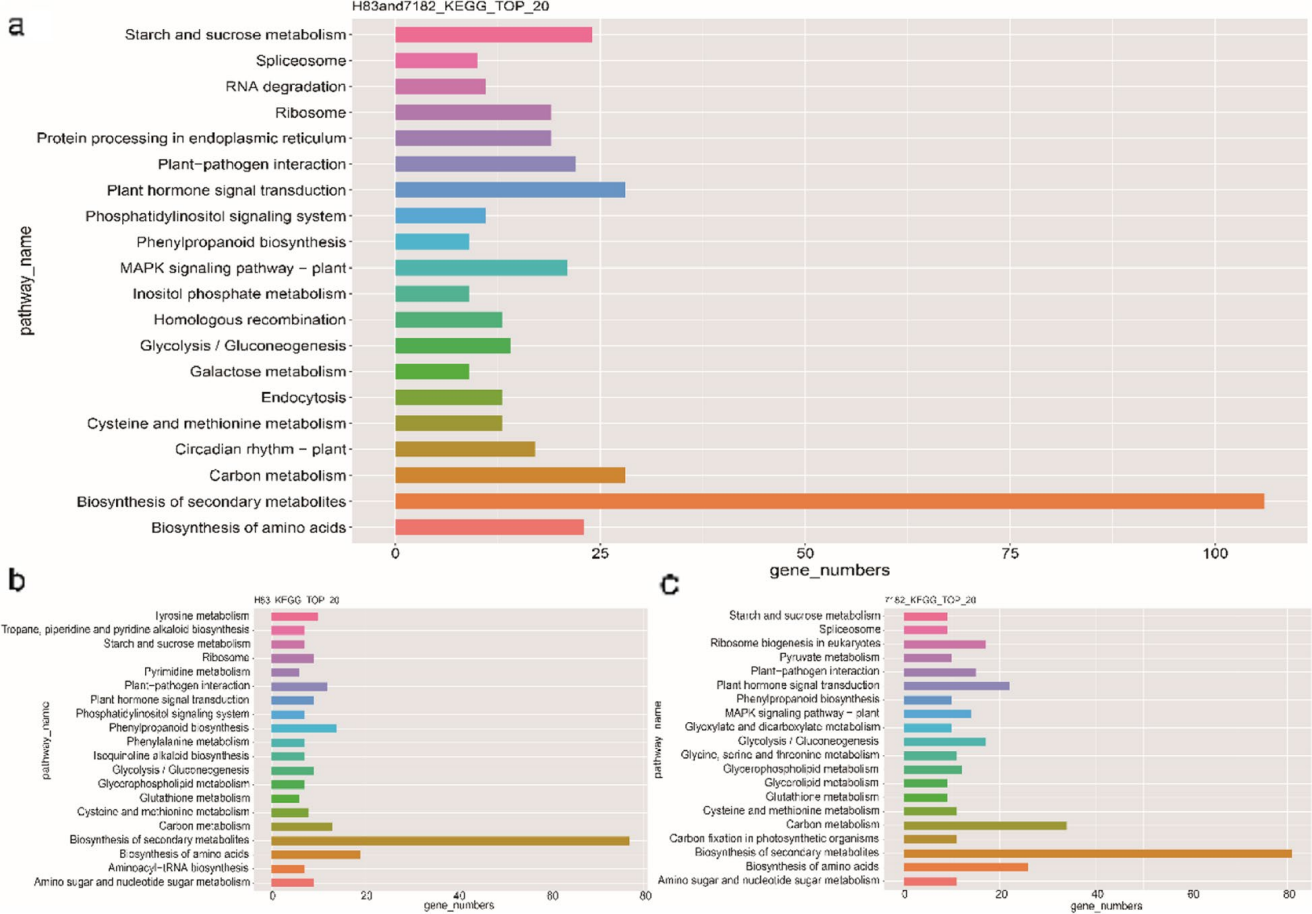
KEGG enrichment analysis of DEGs in resistant material H83 and susceptible material 7182 at different times after inoculation. The x-axis represents the gene ratio. The y-axis represents the KEGG pathways enriched for DEGs. The bubble size indicates the number of DEGs. The color of the bubble indicates the significance of the pathway enriched for DEGs according to the adjusted *P*-value. (**a**–**b**) KEGG classifications of DEGs in susceptible material 7182 at 36 and 72 h post-inoculation, respectively. (**c**–**d**) KEGG classifications of DEGs in resistant material H83 at 36 and 72 h post-inoculation, respectively

Kyoto Encyclopedia of Genes and Genomes (KEGG) pathway enrichment analysis was performed in order to determine the metabolic pathways where the DEGs functioned (the pathways with the highest number of DEGs in the top 20, Fig. 6; details in Additional file 3: Table S3).

The common DEGs in both materials, the specific DEGs in the resistant material H83 and the specific DEGs in the susceptible material 7182 were annotated to 113, 107 and 113 metabolic pathways separately. The metabolic pathways with significant enrichment of these three types of DEGs were all “biosynthesis of secondary metabolites”, “carbon metabolism”, “biosynthesis of amino acids” “plant hormone signal transduction” and “plant-pathogen interaction” pathway. In addition, the specific DEGs unique to the resistant material is also significantly enriched in the “phenylpropanoid biosynthesis”. These results suggest that infection by the plant pathogen led to differential regulation of the expression of genes related to hormone synthesis through signal transmission to modulate the levels of hormones and prevent invasion by the pathogen. On the other hand, after sensing the infection of pathogens, plants resist pathogens by regulating the biosynthesis of secondary metabolites and phenylpropanoid biosynthesis. Amino acid biosynthesis and carbon metabolism pathways provide the energy required for plant growth and development as well as resistance to infection by pathogens. In addition, by analyzing the key metabolic pathways, we summarized some candidate genes for sheath blight resistance in some important metabolic pathways, which are listed in Table 1.

**Table 1:** Candidate genes implicated in the resistance response to *Rhizoctonia cerealis*

gene_description	gene_id	gene_chromosome	gene_strand
	TraesCS3D02G484100	3D	-
NPR1	TraesCS4A02G294400	4A	+
	TraesCS3B02G337700	3B	+
	TraesCS1A02G276600	1A	+
TGA	TraesCS5A02G174200	5A	+
	TraesCS3A02G372400	3A	-
	TraesCS5A02G360200	5A	+
	TraesCS1D02G322000	1D	+
CNGCs	TraesCS6A02G222600	6A	-
	TraesCS5B02G400100	5B	+
	TraesCS2B02G227000	2B	-
	TraesCS2D02G229100	2D	+
	TraesCS5B02G474500	5B	-
	TraesCS6B02G111800	6B	-
CDPK	TraesCS4D02G107200	4D	+
	TraesCS5B02G109300	5B	-
	TraesCS2A02G456100	2A	-
	TraesCS6B02G288600	6B	+
	novel.10485	6D	-
	novel.5221	3D	+
	novel.10225	6D	+
	novel.7873	5B	+
	TraesCS6D02G152100	6D	-
	TraesCS3A02G038300	3A	-
	TraesCS1A02G238800	1A	-
CaM/CML	TraesCS2A02G349100	2A	-
	TraesCS3B02G553900	3B	-
	TraesCS7D02G133400	7D	-
	TraesCS5B02G135100	5B	+
cinnamoyl-CoA reductase	TraesCS5D02G168400	5D	-
	TraesCS5B02G405300	5B	+
peroxidase	TraesCS7D02G347300	7D	-
	TraesCS7D02G315600	7D	-
	TraesCS5A02G205600	5A	+
	TraesCS5B02G209800	5B	+
cinnamyl alcohol dehydrogenase	TraesCS2B02G078600	2B	+
	TraesCS3D02G440900	3D	+
	TraesCS6B02G201600	6B	+
beta-glucosidase 32-like	TraesCS5A02G295400	5A	-
Chalcone synthase	TraesCS6D02G018500	6D	+
Small GTPase	TraesCS4D02G267900	4D	-
SWEET sugar transporter	TraesCS6A02G382400	6A	+
	TraesCS6D02G367300	6D	+
Protein kinase-like domain	TraesCS1D02G017700	1D	-
	TraesCS1D02G017800	1D	+
	TraesCS1B02G022300	1B	-
VQ	TraesCS7B02G233400	7B	+
	TraesCS7D02G329300	7D	+
RNA recognition motif domain	TraesCS2A02G156100	2A	-

### Identification of transcription factors

**Fig. 7:**
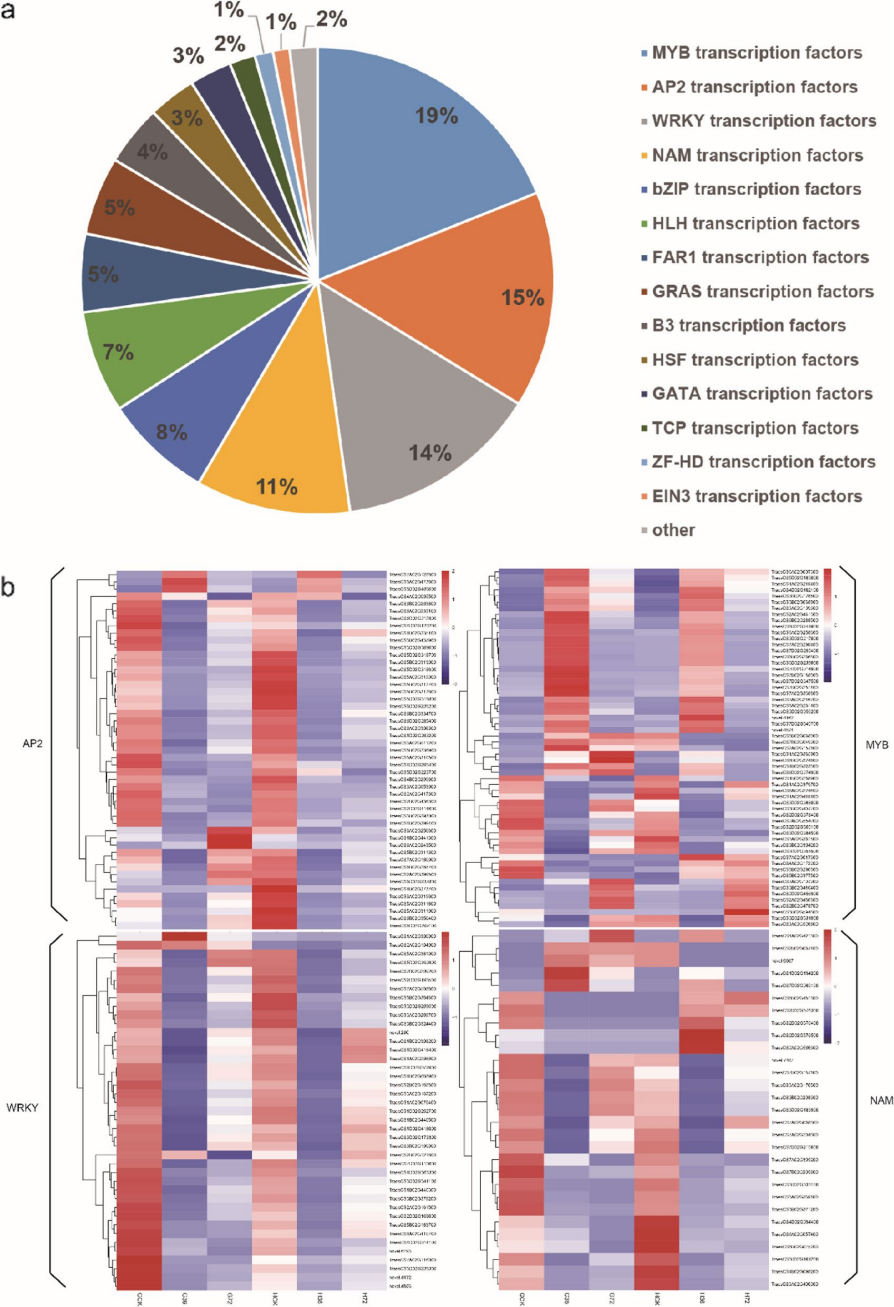
Analysis of specific differentially expressed transcription factors in both materials. Expression levels are shown for the MYB, AP2, WRKY, NAC and transcription factors in resistant material H83 and susceptible material 7182. FPKM values are represented by color gradient of low = navy blue to high = red brick

Transcription factors play important roles in plant disease resistance by regulating genes in signal transduction pathways [34–37]. There are 794 DEGs annotated as transcription factors in this study, which belong to 14 transcription factor families comprising MYB, AP2, WRKY, NAM, bZIP, HLH, FAR1, GRAS, HSF, B3, GATA, TCP, ZF-HD and EIN3 transcription factors. MYB transcription factors were most common and they were encoded by 150 DEGs, which accounted for 19% of the total, followed by AP2 transcription factors (119 DEGs, 15%), WRKY transcription factors (111 DEGs, 14%) and NAM transcription factors (84 DEGs, 11%). The ratios of these transcription factor families are shown in Fig. 7 a. Based on previous studies, we speculate that MYB, AP2, WRKY and NAM may play important role in the infection of *Rhizoctonia cerealis*. Further analysis of MYB, AP2, WRKY and NAM transcription factors showed that 178 were common DEGs in both materials, 139 were unique differentially expressed transcription factors in H83, 147 were unique differentially expressed transcription factors in 7182. Then we performed cluster analysis on the common differential transcription factors in both materials and the unique differential transcription factors of H83 (Fig. 7 b, Fig.S1; details in Additional file 5:Table S5). We found that the expression trend of AP2, WRKY, MYB and NAC family transcription factors shared by the two materials was the same except for a few transcription factors. We found that the expression trend of AP2 and WRKY family transcription factors shared by the two materials was the same except for individual transcription factors. That is, after being infected by *Rhizoctonia cerealis*, most genes showed a continuously down-regulated expression pattern. The expression patterns of individual genes in the two materials were different. For example, TraesCS6A02G256600 and TraesCS1B02G441300 of AP2 family were downregulated in H83 at 36 h post-inoculation, but up-regulated at 72 h post-inoculation, and down-regulated at both inoculation time points in 7182. TraesCS6A02G243500 gene of AP2 transcription factor family and WRKY transcription factors TraesCS1A02G300900, TraesCS2B02G121800 and TraesCS2A02G104900 of WRKY transcription factors family showed the opposite expression trend in both materials. The expression of some transcription factors of MYB and NAM families also showed an opposite trend in the two materials. These results suggest that MYB, AP2, WRKY, and NAC family transcription factors, especially those unique to H83, are common in the two materials, but the transcription factors with inconsistent expression trend in the two materials may play an important role in the resistance of H83 to sheath blight.

### qRT-PCR analysis of candidate genes related to sheath blight resistance in wheat

In order to verify the reliability of the transcriptome sequencing results, we selected 10 DEGs related to wheat sheath blight resistance and analyzed their expression levels at 72 h post-inoculation by qRT-PCR. These genes encoded chalcone synthase (TraesCS6D02G018500), Ras (TraesCS4D02G267900), SWEET sugar transporter (TraesCS6A02G382400 and TraesCS6D02G367300), Pkinase (TraesCS1D02G017700, TraesCS1D02G017800, and TraesCS1B02G022300), VQ (TraesCS7B02G233400 and TraesCS7D02G329300), and RRM_1 (TraesCS2A02G156100). The qRT-PCR results showed that the trends in the expression levels of these randomly selected DEGs were consistent with those determined by transcriptome sequencing (Fig. 9), thereby indicating that the transcriptome sequencing results obtained in this study were reliable.

## Discussion

In recent years, due to the development of “omics,” transcriptome data have been used widely for investigating the interactions between plants and pathogens in order to screen for disease-resistant candidate genes, gene cloning, and to understand the molecular mechanisms associated with fungal pathogen interactions [29]. Cytological observations and analysis of enriched DEGs showed that the infection pressure was much higher in 7182 than H83. We aimed to identify important genes in the plant disease resistance defense response and key metabolic pathways in order to comprehensively characterize the infection response expression profile in wheat to *Rhizoctonia cerealis*. Further analysis of these DEGs will help us to understand the responses of different wheat varieties with resistance to pathogens.

### Identification of sheath blight infection–related DEGs

According to the results of Venn diagram and histogram, the number of DEGs in the susceptible materials 7182 was higher than that in the resistant materials H83 after infection by *Rhizoctonia cerealis*. Previous studies of disease resistant and susceptible rice inoculated with *Magnaporthe oryzae* showed that more DEGs were also identified in the susceptible materials compared with the resistant varieties. the reason for the result may be that the susceptible materials was successfully invaded into the tissue by pathogens, resulting in the complex adaptation mechanism to the lifestyle of *M. oryzae* [33]. Studies have shown that more DEGs are observed in susceptible rice infected by *F. fujikuroi*, and the number of DEG is consistent with the degree of infection of rice varieties by pathogens [32]. In this study, the cytological results show that the susceptible material 7182 is more seriously infected by *Rhizoctonia cerealis* (Fig. 2 G-84h). Therefore, we speculate that the infected susceptible material tissue produces more transcriptional regulatory genes in order to adapt to the lifestyle of *Rhizoctonia cerealis*.

### Phenylpropane biosynthesis and plant disease resistance

Phenylpropane metabolism plays an important role in plant disease resistance and defense response. The main antibacterial substances such as phenols, phytoalexin, lignin and flavonoids need to be synthesized through this pathway. Lignin is the second most abundant polymer on the earth. It mainly accumulates in the cell wall of plants. It not only provides mechanical support for plants, transports water and mineral elements, but also participates in resisting the invasion of pathogens. Cinnamyl alcohol dehydrogenase (CAD) is the key enzyme of monoglycerin biosynthesis before cell wall oxidative polymerization. Studies have shown that CAD 2 is involved in lignin biosynthesis in maize [35, 38] and rice [33], respectively. Recent studies have shown that CAD 12 enhances wheat resistance to *Rhizoctonia cerealis* by regulating the expression of some defense genes and genes related to monomer lignin synthesis [32]. In this study, three upregulated CAD genes were identified in H83. We speculate that the up-regulated of CAD genes in H83 may be related to its resistance to *Rhizoctonia cerealis*. After the monolignols is transported to the cell wall, peroxidases play a role in the process of polymerization of monolignols to form lignin [39]. At the same time, Peroxidases are also PR9 proteins induced by pathogens plant infection, also known as defense protein, which has the function of preventing the spread of pathogens in cells [40–42]. In this study, after being infected by *Rhizoctonia cerealis*, Six up-regulated peroxidases unique to H83 are enriched in phenylpropane metabolic pathway, which is consistent with the previous study that peroxidases were induced to upregulate in rice infected by *Fusarium fujikuroi* [43] Studies have shown that cinnamoyl-CoA reductase is the first rate-limiting enzyme for lignin synthesis [44]. Obviously, if the enzyme synthesis is blocked, it will affect the downstream synthesis pathway of lignin. In this study, the enzyme is specifically up-regulated in H83. Therefore, we speculate that the up-regulated expression of the enzyme may accelerate the downstream pathway of lignin synthesis and promote lignin synthesis, thus playing a positive regulatory role in the resistance to H83. β-glucosidase produces aglycones with higher toxicity to fungal pathogens by hydrolyzing anthocyanins and flavonols [45]. Studies have found that when mango is attacked by *C. gloeosporioides*, the host will secrete β- Glucosidase as part of its defense response [46]. In this study, the upregulation of β- glucosidase may be related to the resistance of H83 to *Rhizoctonia cerealis*. The content of some products and enzyme activity in phenylpropane metabolism are closely related to plant disease resistance. It is generally believed that the content of antibacterial substances and enzyme activity in plants are positively correlated with disease resistance. In conclusion, we speculate that the up-regulated expression of these important genes in phenylpropane pathway may ensure the smooth synthesis of lignin, enhance the synthesis of plant antitoxin and increase the antioxidant capacity of plants, so that H83 obtains the resistance to *Rhizoctonia cerealis* that 7182 does not have.

### Plant–pathogen interactions

Plants have developed their own unique defense systems during long-term evolution with pathogenic microorganisms. It is generally considered that the natural immune system has two levels in plants. Pathogen-associated molecular pattern (PAMP)-triggered immunity (PTI) is the first level, which involves the pattern recognition receptors present on the surfaces of plant cell membranes recognizing various pathogens/microbes. The PAMPs then elicit PTI responses. The second level of the immune response is based mainly on the evolution of resistance genes in plants for recognizing pathogens directly or indirectly, before secreting the corresponding effectors to elicit effector-triggered immunity responses [47]. Chitin is one of the few confirmed PAMPs. When plants interact with pathogens, pattern recognition receptors on the plant cell membrane recognize chitin and chitin oligosaccharides on pathogens to lead to a cascade of responses [48]. In this study, we found that some genes involved in regulating the Ca^2+^ signaling pathway were differentially expressed in response to *Rhizoctonia cerealis* infection (Fig. S2; Additional file 4: Table S4), such as CNGCs, CaM/CML, CDPKs. CNGCs are a family of ligand gated calcium channels in plants and studies have shown that CNGCs are involved in the defense response to pathogens [49]. Loss of function by CNGCs affects the Ca^2+^ signaling pathway involved in the plant defense process [50]. In this study, we found that some CNGC genes were downregulated at 36 h post-inoculation in the susceptible material, but upregulated at 36 h or 72 h post-inoculation in the resistant material. CaM/CML proteins are important sensors during Ca^2+^ signal transduction. Previous studies have shown that CaM/CML expression disorders and functional mutations greatly affected the immune response [51, 52]. Our analysis of plant–pathogen interactions showed that more transcriptional changes in the CaM/CML genes occurred in the susceptible material in response to infection with *Rhizoctonia cerealis* (Additional file 4: Table S4). CDPKs are important receptors during Ca^2+^ signal transduction [53] and they play roles in many physiological processes, including the regulation of biotic stress [54, 55]. In the present study, at 36 h post-inoculation and 72 h post-inoculation, we found that more CDPKs genes were differentially expressed in the susceptible material compared with the resistant material, and most were downregulated (Fig. S2; Additional file 4: Table S4). Thus, we suggest that the downregulation of these genes hindered the interaction with the plant pathogen, thereby resulting in weaker resistance to *Rhizoctonia cerealis* in susceptible wheat line 7182. The relationship between the Ca^2+^ signaling pathway and sheath blight resistance needs further study.

### Plant hormone signal transduction and disease resistance

JA is a fatty acid-derived signaling molecule and it plays an important role in plant disease resistance [56]. JA participates in regulating the defense of plants against necrotic pathogens [57]. Treatment with JA induces the expression of a series of PR genes in rice, and thus JA is involved in immunity and related defense processes against pathogens in rice [58, 59]. In the present study, transcriptome analysis showed that significant changes occurred in the expression of many important genes involved in the JA signal transduction pathway when wheat was infected by *Rhizoctonia cerealis*. Thus, the JA pathway is involved in the resistance to *Rhizoctonia cerealis* in wheat, which is consistent with previous studies of the involvement of the JA signaling pathway in plant resistance to pathogens [32]. COI1 gene is a key regulator in the JA pathway and most JA associated responses are mediated by COI1 [60, 61]. Previous studies have shown that COI1 mutant plants were more sensitive to infection by pathogens [62]. In the present study, COI1 was downregulated in the susceptible material at 36 h and 72 h post-inoculation, but its expression level was normal in the resistant material. JAZ can inhibit the expression of JA response genes by binding to the COI1 receptor protein [63]. Our transcriptome sequencing results showed that JAZ gene expression was disordered, with upregulated (TraesCS2D02G507200) and downregulated (TraesCS7D02G204300) genes at 36 h post-inoculation in the susceptible material. Thus, infection by *Rhizoctonia cerealis* may have blocked the JA signaling pathway in the susceptible material. SA is an important endogenous hormone in the plant response to infection by pathogens [64]. The exogenous application of JA or SA analogues can induce plant immunity to pathogenic bacteria [65, 66]. In the present study, KEGG enrichment analysis of the pathways associated with DEGs showed that some genes with important roles in the response to pathogens were enriched in the SA metabolism pathway, such as NPR1 and TGA. NPR1 is an important regulatory gene in SA-mediated systemic acquired resistance. Previous studies have shown that NPR1 is essential for SA-induced PR gene expression and the defense against pathogens in *Arabidopsis thaliana* [67, 68]. In the present study, the expression of NPR1 was downregulated in the susceptible material at 36 h and 72 h post-inoculation. However, in the resistant material, the expression of NPR1 was downregulated only at 36 h post-inoculation and it returned to the normal level at 72 h post-inoculation. TGA is an important transcription factor in the SA signaling pathway. As-1-like is a cis-acting element that combines with TGA and it is found in several promoter sequences in SA regulatory genes [69]. In the susceptible material, the expression of TGA changed significantly at 36 h and 72 h post-inoculation. By contrast, in the resistant material, the expression of TGA was normal at both time points. Thus, we suggest that the normal expression of key genes in the SA and JA signal transduction pathways is crucial for maintaining resistance to infection by the fungal pathogen. In fact, the SA and JA signaling pathways do not work alone as important signaling molecules in the plant defense response because although they act independently of each other, they are antagonistic at certain points. However, SA–JA crosstalk depends on the type of threat and the order of threat occurrence determines the signaling pathway that takes precedence over the other [69].

### Transcription factors related to hormone regulation and plant disease resistance

Plant transcription factors play important roles in the PTI process and effector-triggered immunity [70, 71]. When plants respond to external stimuli, they activate transcription factors by transduction via a series of signals, which can work alone or with other elements to regulate the expression of downstream defense-related genes, thereby regulating the plant's disease resistance response. MYB is one of the largest transcription factor families in plants. It is well known that MYB proteins are involved in various biological processes, such as seed and flower development, the regulation of primary and secondary metabolism, cell differentiation, and biological stress responses [72]. Previous studies have shown that the overexpression of TaPIMP1, a transcription factor of R2R3-MYB, increased resistance to the hemi-biotrophic pathogen *Bipolaris sorokiniana* in wheat [73]. When wheat was infected by the necrotrophic pathogen *Rhizoctonia cerealis*, the R2R3-MYB transcription factor TaRIM1 regulated the expression of defense-related genes by binding to the MYB-binding site to positively regulate the response to infection by *Rhizoctonia cerealis* [74]. Therefore, MYB transcription factors play important roles in the response to infection by *Rhizoctonia cerealis*. In the present study, we found that the expression levels of some MYB transcription factors changed significantly in the resistant and susceptible materials after infection by *Rhizoctonia cerealis*. The trends in the expression levels of some of these transcription factors were opposite in the resistant and susceptible materials, some were differentially expressed only in resistant materials, and some were differentially expressed only in susceptible materials (supplementary document 5: Table S5). Therefore, it shows that the difference in the expression of MYB transcription factors in the two materials is related to the difference in resistance

The NAC transcription factor family is one of the largest unique transcription factor families in plants and members of this family play important roles in plant growth, development, and stress responses [75]. Studies have shown that many NAC transcription factors are produced in response to infection by pathogens [76]. For example, when rice was infected by *Magnaporthe oryzae*, the expression of *OsNAC19* was increased at the transcriptional level, thereby indicating that *OsNAC19* is involved in the defense response to rice blast fungus [77]. *Magnaporthe oryzae* induced the expression of *OsNAC111* in rice and its overexpression increased the resistance to *Magnaporthe oryzae* [78]. Thus, NAC transcription factors play important roles in resistance to biotrophic pathogens in plant. Recent studies have shown that NAC transcription factors play roles in resistance to necrotrophic fungal diseases. The overexpression of ANAC019/ANAC055 in *Arabidopsis thaliana* attenuated resistance to the necrotrophic fungal pathogen *Botrytis cinerea* by inhibiting the expression of defense related genes [23]. The NAC transcription factor ATAF1 negatively regulates the resistance of *Arabidopsis* to the necrotrophic fungal pathogen *Botrytis cinerea* [79]. The ATAF2 gene, which is highly homologous to ATAF1, is induced by JA and SA. In transgenic plants that overexpress ATAF2, the expression levels of defense genes such as PR1 and PDF1.2 were reduced, and the resistance to *Fusarium oxysporum* decreased. However, the expression levels of these defense genes were increased in loss of function mutants and resistance to the necrotrophic fungal pathogen *Fusarium oxysporum* was also enhanced [80]. Therefore, the NAC transcription factors ATAF1 and ATAF2 in *Arabidopsis* are negative regulators of necrotrophic fungal diseases, and ANAC019/ANAC055 are positive regulators of the necrotrophic pathogen *Botrytis cinerea*. In the present study, we identified eighty-four differential expression NAC transcription factors, where six were upregulated in the resistant material but downregulated in the susceptible material at the two time points, and two were downregulated in the resistant material at two infection time points but upregulated in the susceptible material at the same time points. In addition, thirty-five NAC transcription factors were specifically differential expressed in H83, but did not reach the differential level in 7182. Therefore, we suggest that the NAC transcription factors upregulated in the resistant material may be positive regulators of the necrotizing pathogen *Rhizoctonia cerealis*, whereas the downregulated NAC transcription factors are negative regulators of the pathogen. Further research is needed to understand how members of the NAC transcription factor family might regulate wheat sheath blight.

The WRKY transcription factor family is a large family of plant transcription factors with roles as positive and negative regulators in the plant defense response [70]. A previous study showed that after rice was infected by *Rhizoctonia solani*, the WRKY transcription factor *OsWRKY80* was strongly induced and rapidly expressed, and its overexpression significantly enhanced resistance to *Rhizoctonia solani* [81]. *OsWRKY4* regulates the expression of defense-related genes via the JA and ethylene signaling pathways, thereby activating the defense response to sheath blight in rice [82]. The overexpression of *WRKY30* in rice increases the resistance to *Rhizoctonia solani* by activating the expression of genes related to SA synthesis [83]. In the present study, we identified one hundred and eleven differentially expressed WRKY transcription factors, where two were continuously downregulated in the resistant material at two inoculation time points and one was upregulated and then downregulated, and the trends in the expression levels of these genes were the opposite in the susceptible material relative to those in the resistant material. In addition, thirty-nine WRKY transcription factors were differentially expressed in the resistant material H83, but the expression in the susceptible material 7182 did not reach the significant level. Therefore, we speculate that the differential expression of key transcription factors may be responsible for the different resistance of wheat to *Rhizoctonia cerealis*. These findings provide new ideas for the potential molecular mechanism of wheat resistance to sheath blight

### Analysis of DEGs in important metabolic pathways related to disease resistance

**Fig. 8:**
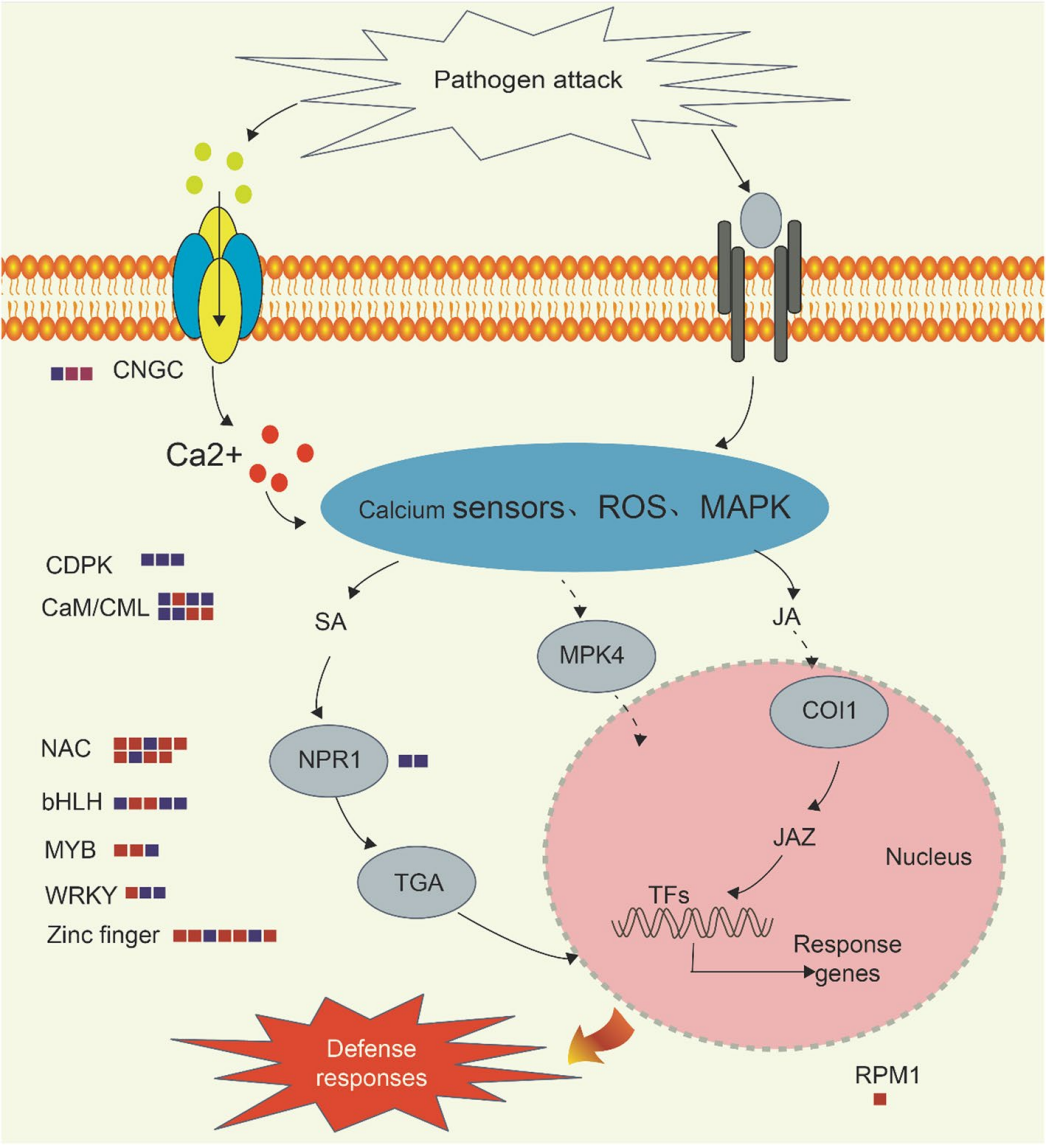
Overview of gene expression and signal transduction in resistant material H83 at 36 h post-inoculation. Each box represents a DEG; Navy blue and red brick colors denote down- and up regulated DEG. The Figure 8 is drawn by me using drawing software (Adobe Illustrator CS5) according to my own experimental results on the basis Fig. 6 b in article of Zhang et al. (2020) [88]. JA: jasmonic acid; SA: salicylic acid (SA); JAZ: jasmonate ZIM-domain; NPR1: Nonexpressor of pathogenesis-related genes 1; CNGCs: cyclic nucleotide gated channels; CaM/CML: calmodulin/calmodulin-like proteins; CDPK: calcium-dependent protein kinase; NPR1: non-expresser of pathogenesis related [PR] 1; TFs: transcription factors; TGA: TGACG motif-binding factor; MAPK: Mitogen-activated protein kinase; COI1: CORONATINE INSENSITIVE1

KEGG enrichment analysis of the DEGs and the analysis of transcription factors showed that plant hormone signal transduction (JA, SA), plant pathogen interaction and transcription factors of differential expression were closely related to disease resistance when the plants were infected by the fungal pathogen. The defense response to necrotrophic fungi in plants is highly dependent on complex signal transduction pathways [84, 85]. Plant hormones such as jasmonic acid (JA) and SA are involved in the defense response to necrotrophic fungi by plants [86]. Ca^2+^ is the simplest messenger, and when the plant is infected by pathogens, the Ca^2+^ channel in the cell will be activated [87]. In order to identify whether the DEGs were involved in these plant disease resistance pathways, we mapped the metabolic pathways regulated by key DEGs (Fig. 8). The COI1 (CORONATINE INSENSITIVE1) gene plays an important role in the JA signal transduction pathway. The COI1 gene encodes an F-box protein and mutations in this gene affect all JA-regulated responses. COI1 combines with the jasmonate ZIM-domain (JAZ) in the presence of JA-Ile and JAZ degrades under the action of the 26S proteasome to release the inhibitory effect on MYC2, thereby initiating the transcription of JA response genes [63, 89]. SA is an important endogenous signaling molecule related to the plant defense response and it plays an important role against biotic stress in plants [90]. Nonexpressor of pathogenesis-related genes 1 (NPR1) and TGA are essential transcription factors in the SA signaling pathway for inducing PR (PATHOGENESIS-RELATED) genes expression and systemic acquired resistance [90]. The regulatory factors and receptor genes expressed in the key signaling pathways in the resistant material H83 and susceptible parent 7182 are shown in Fig. 8 and Additional file 4: Table S4. In the susceptible material 7182, NPR1 (TraesCS3D02G484100), TGA (TraesCS1A02G276600 and TraesCS5A02G174200), COI1 (TraesCS3B02G399200), and JAZ (TraesCS7D02G204300) were downregulated at 36 h post-inoculation, whereas JAZ (TraesCS7D02G204300) was upregulated. NPR1 (TraesCS4A02G294400) and COI1 (TraesCS3B02G399200) were still downregulated at 72 h post-inoculation, whereas TGA (TraesCS3A02G372400) was upregulated. However, in the resistant material H83, only NPR1 (TraesCS4A02G294400 and TraesCS3B02G337700) was downregulated at 36 h post-inoculation, and the expression levels of all the other genes were unchanged. At 72 h post-inoculation, the expression levels of the genes involved in these two pathways were normal. Cyclic nucleotide gated channels (CNGCs), calmodulin/calmodulin-like proteins (CaM/CML), and calcium-dependent protein kinases (CDPKs) play important roles in Ca^2+^ signaling pathways. In this study, all of these genes were differentially expressed in 7182 at 36 h post-inoculation, and most of them were downregulated. At this time, the expression of CNGCs was upregulated, and less CDPK was differentially expressed in the resistant material H83. At 72 h post-inoculation, the expression levels of CNGCs and CDPK were significantly different in the susceptible material. In the resistant material, only CDPK was differentially expressed and CDPK (Traescs5B02G109300) was upregulated. Thus, the expression levels of key transcription factors and regulatory factors in plant hormone signaling pathways and calcium signaling pathways were more stable in the resistant material after infection by the pathogen. Therefore, we suggest that the normal functioning of plant signal transduction pathways might be important for plant resistance to infection by *Rhizoctonia cerealis*.

**Fig. 9:**
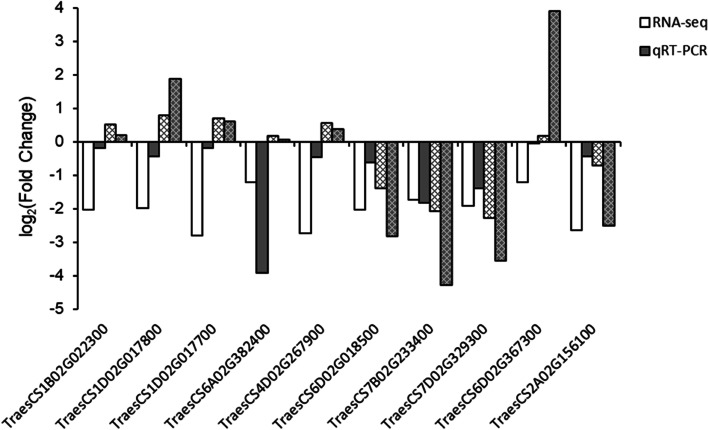
qRT-PCR identification of DEGs obtained from RNA sequencing results. The qRT-PCR values are the averages based on three replicates. Log2 (fold change) represents the logarithm of the gene expression level at 72 h relative to that at 0 h post-inoculation. The grid histogram represents the gene expression data in the susceptible material 7182 and the plain histogram represents the expression data in the resistant material H83

## Conclusions

This study is the first to explore the mechanism of disease resistance between resistant and susceptible wheat by comparative transcriptome, which explored the interaction between resistant/susceptible wheat and *Rhizoctonia cerealis*. Some DEGs related to disease resistance were identified and predicted to be related to plant signal transduction, Ca^2+^ channels and plant pathogen interaction. These results can help us understand the potential mechanism of wheat resistance to *Rhizoctonia cerealis* infection, and provide the basis for the fine mapping and cloning of candidate genes.

## Methods

### Plant materials and inoculation with pathogen

Common wheat 7182 and wheat–*Psathyrostachys huashanica* Keng derived line H83 were used in this study. H83 is an introgression line developed from sesquidiploid line H8911, which was obtained by crossing *Psathyrostachys huashanica* Keng and 7182 as the maternal parent, and backcrossing with 7182 [91, 92]. Previous studies have shown that H83 has inherited the biological characteristic of sheath blight resistance from the male parent *Psathyrostachys huashanica* Keng, which is not present in the female parent 7182 [93]. In the 1980s, Shuyang Chen and Langran Xu, the first researchers in China to cross common wheat and *P. huashanica*, identified and collected wheat wild related material *P. huashanica* [94]; and we have obtained the permission to collect wheat materials used in this study. The *Psathyrostachys huashanica* Keng were taken from Huashan Mountains, Shaanxi Province, China. The wheat materials 7182 were obtained from The National Wheat Improvement Center of China and the wheat-*P. huashanica* derived lines H83 were developed by our laboratory. All materials were deposited in the College of Agronomy, Northwest A & F University, China. The collection and treatment of the experimental materials were in accordance with Wild Plants Protection Regulation of China. The study protocol complied with relevant institutional, national, and international guidelines and legislation. All materials used in this study were cultivated in an artificial climate chamber at Northwest A&F University, Yangling, China. The seeds were vernalized in a vernalization incubator for 30 days until they were budding, before moving them into an artificial climate chamber for further cultivation until the booting stage. During this stage, the photoperiod was set at day/night of 14 h/10 h with temperatures of 18°C/16°C, respectively, and the humidity was set at 70%. Sheath blight strain R0301 was used for inoculation and it was provided by Jiangsu Academy of Agricultural Sciences, China. Before inoculation, strain R0301 was cultured on potato dextrose agar medium covered with toothpicks for about two weeks, before removing toothpicks covered with the white *Rhizoctonia cerealis* fungus. When the wheat plants reached the booting stage, a toothpick covered with fungal hyphae was inserted in the sheath at the connection between the penultimate leaf and stem, before wrapping with sterilized wet absorbent cotton, and the absorbent cotton was kept wet during the experiment. Only the main tillers were inoculated in each individual plant. The temperature was adjusted to 24°C/20°C (day/night) and the humidity was >80% during the inoculation period, with uniform light in this process. Samples were collected of the whole leaves (after 0 h, 12 h, 24 h, 36 h, 48 h, 60 h, 84 h, 96 h, and 120 h) and corresponding leaf sheaths (after 0 h, 12 h, 24 h, 36 h, 48 h, 60 h, 84 h, 96 h, and 120 h) of the penultimate leaf of inoculated and non-inoculated plants. Three biological replicates were taken at each time point, and each biological replicate randomly collected three penultimate leaves of inoculated or non-inoculated plants. Wheat materials H83 and 7182 were planted in 10 pots with 10 plants in each pot. All plants were planted in parallel. The leaves were rinsed with distilled water and placed into cryopreservation tubes with a volume of 5 mL, before freezing rapidly in liquid nitrogen and storing in an ultra-low temperature refrigerator at –80°C for subsequent RNA extraction. The leaf sheath was cut into 0.5 cm-long segments with a double-sided blade and fixed in glutaraldehyde fixative solution for subsequent cytological sample preparation.

### Phenotypic changes and cytological observations

Expansion of *Rhizoctonia cerealis* on the wheat leaf sheath was observed with a Nova Nano SEM-450 scanning electron microscope and photographed. The leaf sheath treatment process was conducted basically as described by Xu et al. [95] and Zhang et al. [96]. To observe the fungal infection process on wheat leaf sheaths, semi-thin and ultra-thin sections of the leaf sheaths were fixed, dehydrated, infiltrated, and embedded according to Yang et al. [97], and stained as described by Wang et al. [98]. Semi-thin cross sections with a thickness of 1 μm were stained with toluidine blue and observed by a microscope with Axio Imager A2. Observation and photography of ultrathin sections were conducted using a TECNAI G2 SPIRIT BIO transmission electron microscope (FEI Company, Hillsboro, OR, USA).

### RNA extraction, library construction, and RNA sequencing

Total RNA was extracted from 18 samples of materials 7182 and H83 after three treatment periods (0 h, 36 h, and 72 h), with three biological replicates for each sample. The integrity of the RNA and contamination were monitored by agarose electrophoresis. The purity and integrity of the RNA were detected using a nanophotometer spectrophotometer (IMPLEN, CA, USA) and RNA Nano 6000 Assay Kit for the Bioanalyzer 2100 system (Agilent Technologies, CA, USA), respectively. Sequencing libraries were generated using a NEBNext® UltraTM RNA Library Prep Kit for Illumina® (NEB, USA) according to the manufacturer’s recommendations, and index codes were added to attribute sequences to each sample. Next, eukaryotic mRNA was enriched using Oligo (dT) magnetic beads. The mRNA obtained was randomly interrupted by a divalent cation in NEB Fragmentation Buffer. The first strand of cDNA was synthesized using the M-MuLV reverse transcriptase system with fragment mRNA as the template and random oligonucleotides as primers. RNA strands were degraded with RNase H and the second strand of cDNA was synthesized with dNTPs as raw materials in the DNA polymerase I system. The purified double-stranded cDNA was subjected to terminal repair, before adding poly (A) tails and ligating to the sequencing adapter, and AMPure XP beads (Beckman Coulter, Beverly, USA) were used to screen cDNA sequences of 250–300 bp for amplification by PCR. The PCR products were purified using AMPure XP beads and the quality of the library was assessed with the Agilent Bioanalyzer 2100 system before sequencing using the Illumina HiSeq™2500 system at Novogene Biotech Co. Ltd (Tianjin, China).

### Sequence alignment

The original data obtained by sequencing contained sequencing adapters or low quality reads. In order to ensure the quality and reliability of the analysis, the original data were filtered, which mainly involved removing reads with adapters, as well as reads with N (N indicates that the base information cannot be determined) and low-quality reads (qphred ≤ 20). Q20, Q30, and the GC contents were calculated for the clean data. All subsequent analyses were conducted based on the clean data. The clean reads were rapidly and accurately mapped to the IWGSC RefSeq 1.1 reference genome to obtain positioning information for the reads in the reference genome by using HISAT2 (v2.0.5), and the mapping parameters are hisat2 -x faindex -p 4 --dta -t --phred33 -1*_1.clean.fq.gz -2 *1.unmap.fq.gz --un-conc-gz *.unmap.fq.gz 2>*log|samtools sort -O BAM --threads 4 -o *.bam -. If these reads were mapped to the genome, they will be retained, otherwise they will be removed. New transcripts were assembled using StringTie (v1.3.3b) [99] which applied network flow algorithm and optional de novo assembly to splice transcripts. The parameters used for the prediction of new transcripts are stringtie <bam> -p 4 -G <gtf> -o <out_gtf>. The specific transcriptome analysis process and the assembly process of new genes are shown in Supporting Information S1. The new transcripts were annotated with Pfam, SUPERFAMILY, Gene Ontology (GO), Kyoto Encyclopedia of Genes and Genomes (KEGG), and other databases. The featureCounts (v1.5.0-p3) program was used to calculate the expected number of fragments per kilobase of transcripts per million mapped reads.

### Bioinformatics analysis of differentially expressed genes (DEGs)

Differential expression analysis was performed for the combinations tested (three biological replicates per group) using the DESeq2 R package (v1.16.1), which provides a statistical program for models based on a negative binomial distribution to determine differential expression in digital gene expression data. The resulting *P*-values were adjusted using Benjamini and Hochberg’s approach for controlling the false discovery rate. Genes with an adjusted *P*-value <0.01 and |log2foldchange| > 1 found by DESeq2 were assigned as DEGs. The clusterProfiler R package (v3.4.4) was used for GO enrichment analysis of DEGs, where the deviation in the gene length was corrected. GO terms with a corrected *P*-value<0.05 were considered significantly enriched DEGs. KEGG (http://www.genome.jp/kegg/genes.html) is a database resource for understanding advanced functions and utilities in biological systems, such as cells, organisms, and ecosystems, based on molecular level information, especially the large-scale molecular data sets generated by genome sequencing and other high-throughput databases [100]. The clusterProfiler (v3.4.4) program was used to statistically analyze the enrichment of DEGs in KEGG pathways. KEGG pathway enrichment with a corrected *P*-value<0.05 were considered significantly enriched DEGs. Blast2GO was used to classify the DEGs.

### Analysis of expression profiles of candidate resistance genes by qRT-PCR

Eighteen RNA samples were extracted from the two materials (7182 and H83) at three times post-infection (0 h, 36 h, and 72 h) according to the instructions provided with the RNAsimple Total RNA kit (TIANGEN). The RNA was then reverse transcribed using a Reverse Transcription Kit with gDNA Eraser. The specific primers for qRT-PCR were designed with Primer5 and tested with Primer-BLAST (https://www.ncbi.nlm.nih.gov/tools/primer-blast/primertool.cgi). Actin was used as a standardized internal reference gene to determine gene expression levels. The primers used in this experiment were synthesized by Xi'an Qingke Biotechnology Co. Ltd., China and they are described in Additional file 6: Table S6. qRT-PCR was performed using the QuantStudioTM Real-Time PCR System. Three biological replicates were performed for each biological replicates. The expression levels of the candidate resistance genes were determined with the 2–ΔΔCt method.

## Supplementary Information

**Additional file 1:** Table S1.

**Additional file 2:** Table S2.

**Additional file 3:** Table S3.

**Additional file 4:** Table S4.

**Additional file 5:** Table S5.

**Additional file 6:** Table S6.

**Additional file 7:** Fig. S1.

**Additional file 8:** Fig. S2.

**Additional file 9: **Supplementary material.

## Data Availability

All related plant materials are available and comply Wild Plants Protection Regulation of China. The datasets supporting the conclusions of this article are included within the article and its supplementary files. We have deposited our raw sequencing data in NCBI SRA database, the accession number for our submission is: PRJNA749387. My SRA records will be accessible with the following link after the indicated release date: https://www.ncbi.nlm.nih.gov/sra/PRJNA749387.

## Abbreviations

DEGs: differentially expressed genes;
QTL: quantitative trait loci
JA: jasmonic acid
SA: salicylic acid (SA)
JAZ: jasmonate ZIM-domain
NPR1: Nonexpressor of pathogenesis-related genes 1
CNGCs: cyclic nucleotide gated channels
CaM/CML: calmodulin/calmodulin-like proteins
CDPKs: calcium-dependent protein kinases
NPR1: non-expresser of pathogenesis related [PR] 1
TFs: transcription factors
TGA: TGACG motif-binding factor
MAPK: Mitogen-activated protein kinase
COI1: CORONATINE INSENSITIVE1
PAMP: Pathogen-associated molecular pattern
PTI: (PAMP)-triggered immunity
PR: PATHOGENESIS-RELATED
GO: Gene Ontology (GO)
KEGG: Kyoto Encyclopedia of Genes and Genomes
