# Supplementary material for: Comparative transcriptome analysis of resistant and susceptible wheat in response to Rhizoctonia cerealis
Source: BMC Plant Biol. 2022 May 10;22:235. doi: 10.1186/s12870-022-03584-y (PMC9087934; doi:10.1186/s12870-022-03584-y)
Supplement: Supplementary file 7 — Additional file 7: Fig. S1. [file 12870_2022_3584_MOESM7_ESM.docx]

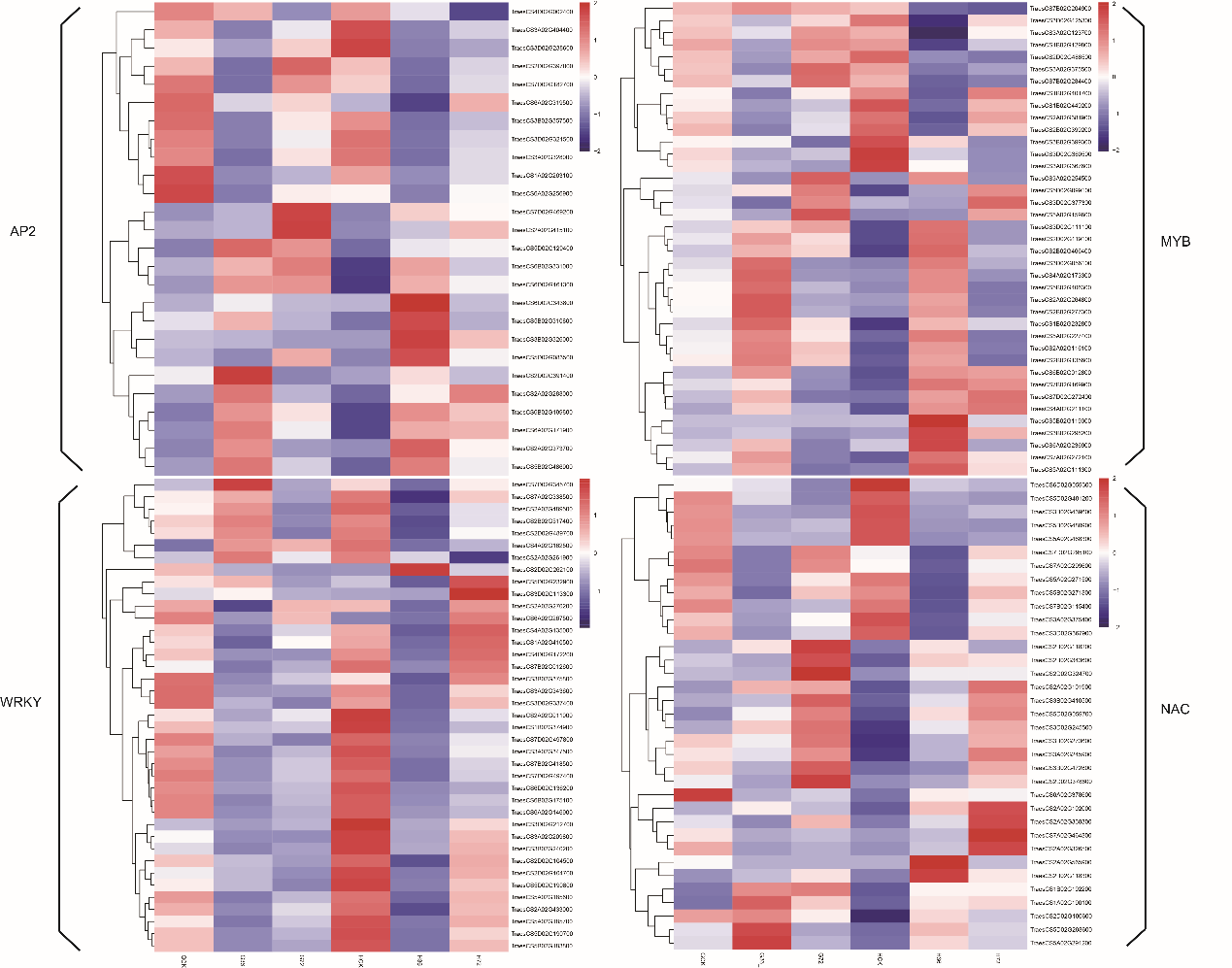


**Fig. S1** Analysis of specific differentially expressed transcription factors in resistant material H83. Expression levels are shown for the MYB, AP2, WRKY, NAC and transcription factors in resistant material H83 and susceptible material 7182. FPKM values are represented by color gradient of low = navy blue to high = red brick.
