# Supplementary material for: Comparative transcriptome analysis of resistant and susceptible wheat in response to Rhizoctonia cerealis
Source: BMC Plant Biol. 2022 May 10;22:235. doi: 10.1186/s12870-022-03584-y (PMC9087934; doi:10.1186/s12870-022-03584-y)
Supplement: Supplementary file 8 — Additional file 8: Fig. S2. [file 12870_2022_3584_MOESM8_ESM.docx]

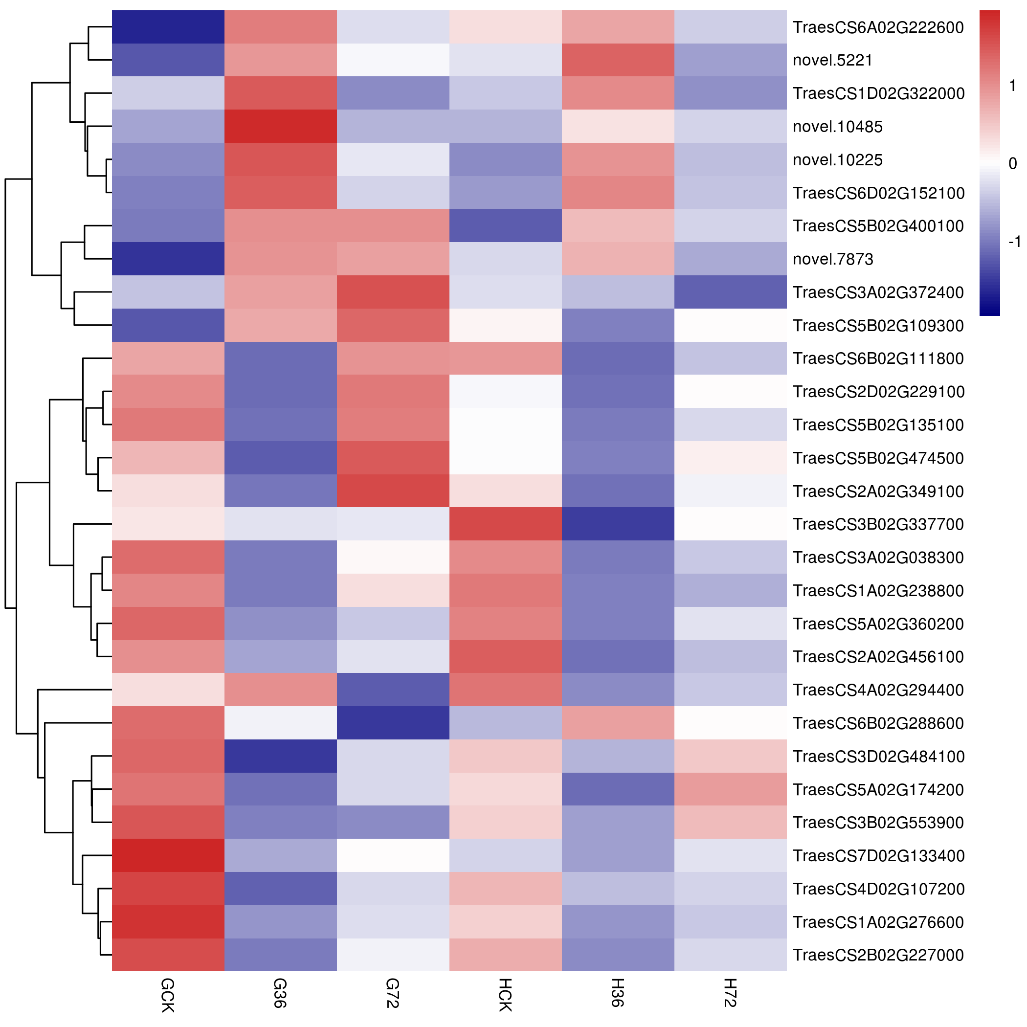


Fig. S2 Heat map analysis of differentially expressed genes in important metabolic pathways.

DEGs involved in pathways. FPKM values are represented by color gradient of low = navy blue to high = red brick.
