# Supplementary material for: Comparative transcriptome analysis of resistant and susceptible wheat in response to Rhizoctonia cerealis
Source: BMC Plant Biol. 2022 May 10;22:235. doi: 10.1186/s12870-022-03584-y (PMC9087934; doi:10.1186/s12870-022-03584-y)
Supplement: Supplementary file 9 — Additional file 9: Supplementary material. [file 12870_2022_3584_MOESM9_ESM.docx]

**Supporting Information S1:** **Transcriptome analysis and transcript assembly process**

***The whole transcriptome analysis process is as follows:**

1. The reads were matched to the reference genome using HISAT.
2. The reads compared with the reference genome will be presented to stringtie for transcript assembly. Stringtie will assemble each sample separately. During the assembly process, the expression level of each gene and isoform will be estimated.
3. All transcripts are presented to the merge function of stringtie for merge.
4. The merge data is presented to stringtie again. Stringtie uses the merge data to re estimate the abundance of transcripts, and can also provide additional data on the number of transcripts reads to Balltown in the next step.
5. Balltown obtained all transcripts and their abundances from the previous step, and classified and counted them according to the experimental conditions.

***Assembly details are as follows:**

**1 、assembly**

The transcriptome was assembled by software stringtie. Firstly, we generate a gtf file for each bam file, which mainly records the assembly information of transcripts.

**2、merge**

Then, gtf files containing transcript information are combined into one gtf by using software stringtie.

**3、gffcompare**—Compare with known transcript annotation files

To compare the quantitative information of transcripts of different samples, the transcripts need to be stored in the same format. Generally, the output result of assembly software is gtf or gff. Since a large number of new transcripts are generated during the assembly process, and it is obvious that we can’t clarify the biological significance by observing the only annotation information -- the starting position on the chromosome, we need to compare them with the known transcripts annotation file -- annotation.gtf, So as to establish a link between the newly obtained transcripts and the annotated transcripts, so that we can better find new transcripts.

**4 、New gene screening**

When screening new transcripts, start with the class codes of the GTF file, which records the location information of each transcript relative to the known transcript. Through this class_code generally, three types of transcripts are selected, namely:

i: transcript of intron region

u: New transcripts of intergenic region

x: Antisense chain transcripts of known exons

**5 、format conversion**

The GFF or GTF format file of the new gene is generated by reading the gffcmp.annotated.gtf file, and the GFF file is transformed into the corresponding transcript sequence by reading the reference sequence
**6 、Transdecoder predicts CDS**

Identify the potential coding region in the transcript sequence, that is, predict CDS.

**7、Filter**

By judging the ORF length in the protein sequence, set a threshold of 50 to filter out those new genes encoding shorter proteins and reduce false positives.

8、**Merge known and new gene GTF files.**
